# Immature Gastric Teratoma in a Newborn: A Case Report 

**Published:** 2016-04-10

**Authors:** Sanjay Kumar, Hemant Yadav, Kamal Nain Rattan, Divya Srivastava, Abha Chandana, Sant Prakash

**Affiliations:** 1 Department of Pathology, PGIMS, Rohtak; 2 Department of Paediatric Surgery, PGIMS, Rohtak

**Keywords:** Teratoma, Immature, Gastric

## Abstract

A case of immature gastric teratoma in a neonate is being reported here. The neonate was presented with abdominal mass and distension and managed with excision of mass; the patient is doing fine postoperatively.

## CASE REPORT

A 15-day-oldmale child was brought to the hospital with complaint of abdominal distention and a palpable mass in left upper abdomen since birth. There were no other generalised or systemic complaints. The child was born through a full term vaginal delivery and cried immediately after birth. Child was on breast feed and there was no history of exposure to any drugs or radiation to the mother in the ante-natal period. Abdominal examination revealed a firm, irregular mass in left upper abdomen. Complete blood count revealed Hb of 18.0 g/dL and total leukocyte count of 11,500/cm3. Other biochemical investigations were within normal limits. Ultrasonography revealed a solid-cystic mass with mixed echogenicity in left upper abdomen and continuous with gastric outline. Computed tomography (CT) demonstrated a large, lobulated, heterogeneous, exogastric mass attached to larger curvature of stomach with solid and cystic components with calcification and fat attenuation, suggestive of teratoma (Fig. 1). An apparent invasion to surrounding structures was not seen. Exploratory laparotomy was performed which revealed a large solid-cystic mass (9.5x5x3cm) arising from postero-inferior wall of the stomach along its greater curvature. The mass was lying outside the stomach without any extension into the lumen of the stomach. The mass was excised en-block and the defect was repaired. The postoperative period was uneventful and child was discharged on 7th postoperative day. The child is still on follow up with serum alpha-feto protein level and is doing well. Histopathological examination showed presence of stratified squamous epithelium, dermal appendages, gut mucosa, respiratory lining, and cartilage along with multifocal aggregates of immature neuro-ectodermal tissue, suggestive of immature gastric teratoma. 

**Figure:**
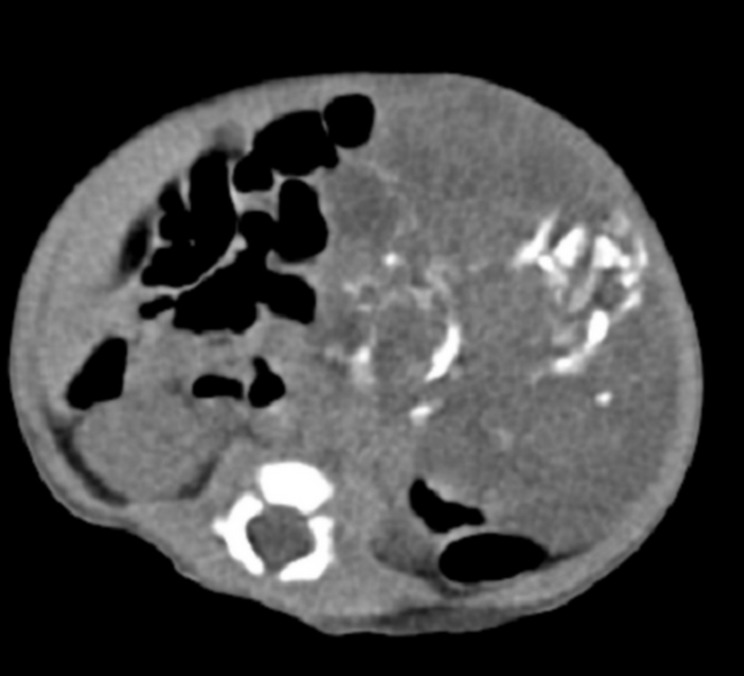
Figure 1: Computerized Tomography imaging showing a heterogenous mass with solid and cystic components along with internal calcification.

## DISCUSSION

Teratoma is the most frequent tumor among germ cell neoplasms in children.[1-4]In infancy and childhood the commonest site of teratoma are sacrococcygeal region (60-65%), gonadal (10-20%), mediastinal (5-10%), presacral (5%) and rarely intracranial, retroperitoneal and cervical.[5]There is a striking male predominance of gastric teratoma with only seven cases (7%) occurring in females.[4]In most of the cases, the chief complaints are abdominal distention and lump but sometimes respiratory difficulty can be caused by upward displacement of the diaphragm by the tumor. Some infants can present with vomiting, hematemesis or malena because of ulceration of overlying mucosa in cases of endogastric component.[2] Although gastric teratoma can arise from any part of the stomach, common sites are the lesser curvature of stomach, antrum and fundus of stomach along the posterior wall. Majority of gastric teratoma are exogastric representing approximately 60% of the cases while endogastric growths are present in about 30% of the cases. Mixed exogastric and endogastric growths are rare.[2]In our case the mass was exogastric without any endogastric component attached to greater curvature. Differential diagnosis in paediatric age group should include neuroblastoma, Wilms tumor, hepatoblastoma, rhabdomyosarcoma, liposarcoma, and retroperitoneal teratoma. Histopathologically gastric teratoma can be mature or immature based on the presence and differentiation of neuroglial tissue. Mature gastric teratoma contains mature glial tissue along with other derivatives of all germinal layers and vice versa. Prognosis is excellent as complete excision with primary closure of gastric wall defect give recurrence free survival without adjuvant chemotherapy and radiotherapy. But few cases of immature teratoma with malignant transformation have been described in the literature so regular follow up with abdominal USG and serum AFP is advised in each case.[1]

## Footnotes

**Source of Support:** Nil

**Conflict of Interest:** Nil
